# Over‐the‐wire insertion of a naso‐duodenal feeding tube in a critically ill infant

**DOI:** 10.1002/jpr3.70122

**Published:** 2025-11-27

**Authors:** Hashim Abdul‐Khaliq, Jochen Pfeifer

**Affiliations:** ^1^ Department of Pediatric Cardiology Saarland University Medical Center Homburg Germany

**Keywords:** enteral, guide wire, neonate, nutrition

In a 7‐month‐old boy (body weight 7 kg) with congenital heart disease, short bowel syndrome, and gastric motility disorder, conventional insertion of a duodenal feeding tube was unsuccessful (Figure 1). Alternatively, a catheter‐based method under fluoroscopy was performed. An angiography catheter (4F multipurpose special; Cordis Corp., Miami Lakes, FL, USA) was inserted nasogastrically into the stomach, then a guide wire (0.014'' Whisper™ ES; Abbott Medical, Santa Clara, CA, USA) was advanced into the duodenum over which the catheter could be pushed (Figure 2A). The course of the duodenum then was visualized by manual contrast injection (Figure 2B). Next, a more stable guide wire (0.018'' Nitrex™; ev3 Inc., Plymouth, MN, USA) was carefully inserted into the distal duodenum. To enable an insertion over‐the‐wire, the closed tip of an enteral feeding tube (8 F Freka™ Tube; Fresenius Kabi AG, Bad Homburg, Germany) was cut off. To deburr it, the tip was ground down and washed. Using ultrasound gel to improve gliding ability, the tube could be easily advanced into the duodenum via the wire without any complications (Figure 2C).

**Figure 1:**
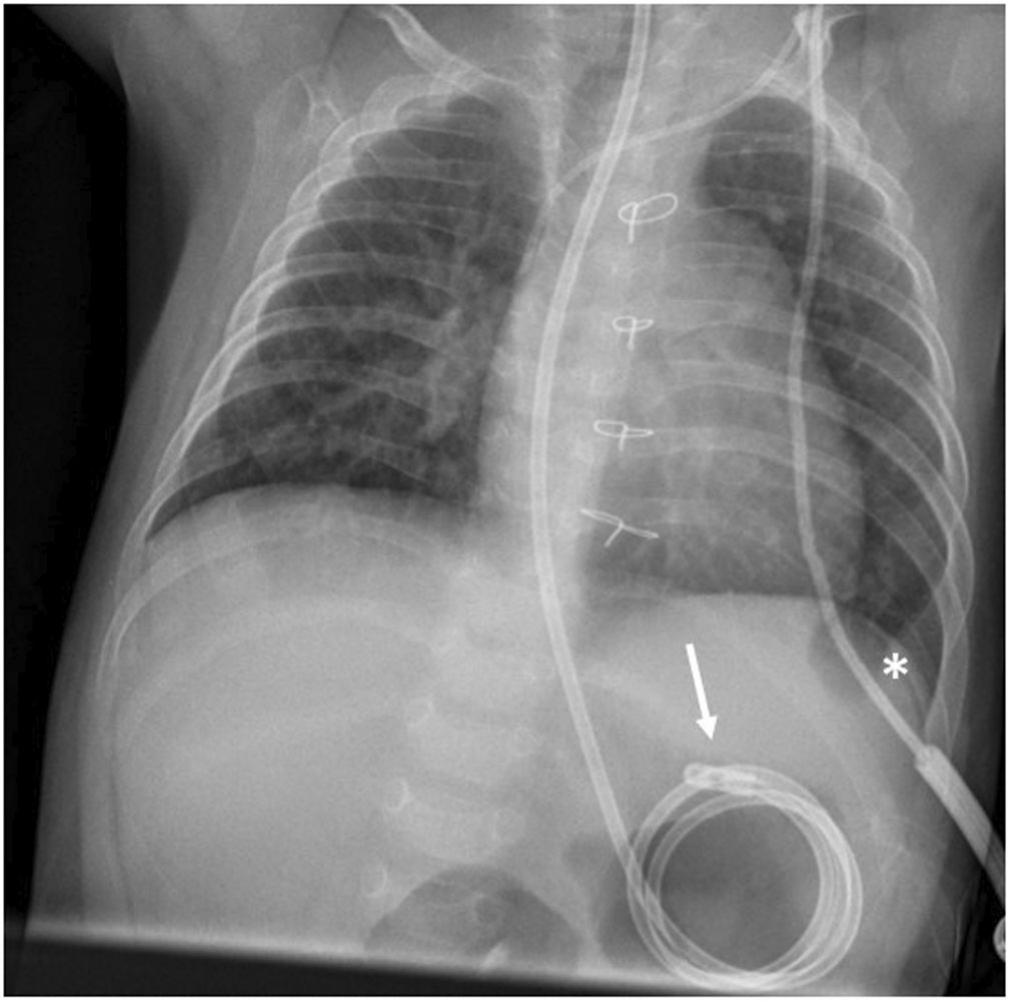
X‐ray of the chest and upper abdomen (posterior‐anterior projection) showing the blindly placed feeding tube, which is coiled up in the stomach instead of being advanced into the duodenum (arrow). A permanent central venous catheter (Broviac™) is marked by an asterisk.

**Figure 2:**
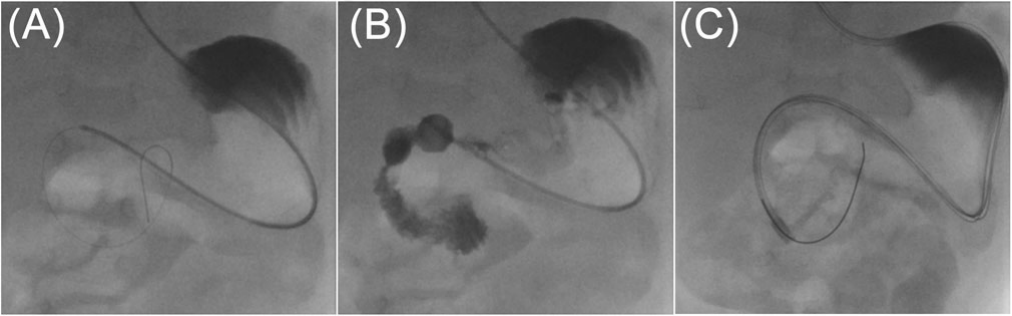
Fluoroscopic depiction (posterior‐anterior projection) of the over‐the‐wire insertion of the naso‐duodenal feeding tube: (A) guide wire and catheter are advanced into the duodenum; (B) contrast image showing the position of the duodenum; (C) the feeding tube is advanced into the duodenum via a stable guide wire.

Neonates and infants with severe diseases often experience nutrition difficulties. The use of naso‐gastric or, in case of gastric emptying disorders, post‐pyloric tubes may be necessary.^1^ Blind bedside placement of duodenal tubes can be challenging.^2^ Endoscopic or percutaneous approaches are alternative, but invasive techniques. Derived from conventional cardiovascular catheterization using angiography catheters under brief fluoroscopy, the over‐the‐wire technique is a simple method for the safe and correct placement of duodenal tubes.^3^, ^4^

## CONFLICT OF INTEREST STATEMENT

The authors declare no conflicts of interest.

## ETHICS STATEMENT

Written informed consent was obtained from the patient's father for publication of the images, and any potentially identifying information has been removed.
